# Care planning at home: a way to increase the influence of older people?

**DOI:** 10.5334/ijic.817

**Published:** 2012-08-10

**Authors:** Helene Berglund, Anna Dunér, Staffan Blomberg, Karin Kjellgren

**Affiliations:** Institute of Health and Care Sciences, Sahlgrenska Academy at the University of Gothenburg, SE-405 30 Gothenburg, Sweden; Vårdalinstitutet, Swedish Institute for Health Sciences, Box 187, SE-221 00 Lund, Sweden; Department of Social Work, University of Gothenburg, SE-405 30 Gothenburg, Sweden; Vårdalinstitutet, Swedish Institute for Health Sciences, Box 187, SE-221 00 Lund, Sweden; Department of Social Work, Lund University, SE-221 00 Lund, Sweden; Vårdalinstitutet, Swedish Institute for Health Sciences, Box 187, SE-221 00 Lund, Sweden; Institute of Health and Care Sciences, Sahlgrenska Academy at the University of Gothenburg, SE-405 30 Gothenburg, Sweden; Department of Medical and Health Sciences, Linköping University, SE-581 83 Linköping, Sweden

**Keywords:** care planning, continuum of care, influence, involvement, older people, qualitative content analysis

## Abstract

**Introduction:** Care-planning meetings represent a common method of needs assessment and decision-making practices in elderly care. Older people’s influence is an important and required aspect of these practices. This study’s objective was to describe and analyse older people’s influence on care-planning meetings at home and in hospital.

**Methods:** Ten care-planning meetings were audio-recorded in the older people’s homes and nine were recorded in hospital. The study is part of a project including a comprehensive continuum-of-care model. A qualitative content analysis was performed.

**Results:** Care-planning meetings at home appeared to enable older people’s involvement in the discussions. Fewer people participated in the meetings at home and there was less parallel talking. Unrelated to the place of the care-planning meeting, the older people were able to influence concerns relating to the amount of care/service and the choice of provider. However, they were not able to influence the way the help should be provided or organised.

**Conclusion:** Planning care at home indicated an increase in involvement on the part of the older people, but this does not appear to be enough to obtain any real influence. Our findings call for attention to be paid to older people’s opportunities to receive care and services according to their individual needs and their potential for influencing their day-to-day provision of care and service.

## Introduction

There is unanimous agreement in health- and social-care legislation, as well as in public and professional opinion, that users have the right to influence planning, decision-making and delivery of health and social care [1–4]. In recent years increasing interest has been focused on older people’s opportunities to choose between different care and service providers [5, 6]. Giving older people more choice and a ‘louder’ voice [7] and encouraging them to assess their own needs [8] are essential parts of the policy of personalisation.

There is, however, a gap between ideal and reality regarding needs assessment and decision-making practices [7, 9–14]. Hardy et al. [9] showed that choice had increased at the strategic level in terms of sector and providers of services but was limited when it came to the day-to-day working of their services. Tension was found in the assessment process at the service user/professional interface associated with the organisational context and broader service environment [12]. Lymbery [7] suggested that problems with the strategy of personalisation relate to the unresolved tensions between needs, rights and resources, such as the contradictions between targeting the resources at those most in need and, conversely, helping people to be independent by focusing on early intervention. In addition, older people are not always able to make choices and take control of their own lives, as intended in the current policy visions [7].

By comparison with other countries, elderly care in Sweden is traditionally described as an extensive, tax-funded high-quality service available to all citizens in need, and is not contingent on means or a lack thereof [15]. Current policy is that older people should be able to live independently, managing with formal and informal care as long as possible [16]. The autonomy of local governments regarding the provision of care and an emphasis on home-based care services represents other characteristics [17]. However, during the 1990s, home-help services provision in particular became increasingly restrictive and standardised. This development has led to a reduced opportunity for the professionals, especially social workers, to consider the older people’s individual situations. In the municipalities, rules and guidelines for needs assessment have led to increased organisational control, and professionals tend to adjust the needs of the older people to existing care services [10, 18, 19]. Within home nursing care, medical needs are not always met, due to local differences relating to examination and treatment access in the patients’ homes [20].

Care planning often provides the context for needs assessment and decision-making. It is a process in which the professionals co-ordinate the planning of future care, in interaction with the older person. The care-planning meeting generally takes place in hospital, prior to discharge. Several professionals participate, from both the hospital and the municipality. The older persons’ possibilities to influence decisions made at those meetings have direct consequences for their everyday lives. The meetings between older people and professionals deciding upon care and services are characterised by an unequal distribution of power [10, 21, 22]. Many studies have pointed out the limited involvement of older people in this context [23–26]. In many cases the professionals dominate the communication [27, 28] and approaches that encourage older people to express their personal wishes are not always satisfactory [11]. The older people’s perspective is filtered out in the assessment process and care routines tend to exclude both the older people and professionals from active decision-making [29]. Another study indicated that older people perceived no actual influence over the decisions about their home help/care [30].

Older people with extensive and complex needs often meet many different care providing organisations and professional groups, making it necessary to have a high degree of coordination and integration. In front-line practice, care-planning meetings with different professionals in health/social care and rehabilitation is regarded as a means to reach a comprehensive assessment of the needs of older care recipients [31–34]. However, the professionals are in the majority and interprofessional working may be construed as a way to increase their power [35]. The professionals may form alliances to convince the older person to accept their interpretations of the situation and accept proposed care and services [35, 36]. Thus care-planning meetings intended to ensure integrated care to older people, may in fact jeopardise their influence and control over decisions vital to their everyday life.

We use the term ‘influence’ to denote the capacity to have an effect on the character or behaviour of someone or something. The tensions involved in promoting older people’s influence on care planning relate to the concepts of people processing, power and empowerment. In human service organisations, such as municipal health and social care for older people, simplification and standardisation of the older people’s needs adjusted to available care and service takes place. In the process of transforming individuals into clients or patients, the professionals collect and assess information about the persons in order to typify them and put them into predetermined categories of needs and corresponding services [37]. Participants’ influence on care planning relates to issues associated with power, status and dependence. The older people’s dependence makes the relationship between them and the professionals asymmetrical [38–40]. Additionally a moral dimension including behaviour rules contributes to the assymetric relations [41]. The ‘frame’ implies that what goes on in interactions between people is governed by unstated rules set by the character of the situation in which the interactions occur [36]. In this case the care-planning meeting may be understood as the actual situation where this frame appears. Some of the actors (i.e., the professionals) define the situation and proper way to act at the care-planning meeting.

In a shift towards more symmetrical relationships, the professionals are believed to give power or ability to the older people in a process of empowerment [13, 42, 43]. Different approaches to empowerment can be identified: a consumerist model in which people are given a choice between professionally defined services, a liberation model relating to the position of oppressed groups within society and a view of empowerment as a professional practice [43].

The organisation of care-planning meetings might have an impact on older people’s possibilities of influence over the decision-making processes. Alternative working methods, such as holding care-planning meetings in the older people’s homes, have been introduced in some cases. How the interactions between the older people and the professionals are affected by where meetings take place have not yet, to our knowledge, been investigated. The overall aim of this study was to describe and analyse older people’s influence on care-planning meetings at home and in hospital. The specific research aims were to:

- characterise the management of the care-planning meetings
- describe the content of initiatives, discussions and decisions in the care-planning meetings
- describe similarities and dissimilarities between care-planning meetings at home and in hospital.

## Methods

### Study context

The study is part of a larger multidisciplinary intervention project entitled “Continuum of Care, From the Emergency Ward to Living at Home—Implementation and Evaluation of an Intervention for Frail Elderly People” [44], performed at the hospital, primary care and municipal care in a city with approximately 60,000 inhabitants on the west coast of Sweden.

A new model of the continuum of care was introduced, including several components described as integrated care strategies, such as case management, interprofessional teamwork and support for older people and their relatives [32, 45]. An assessment of the need for health/social care and rehabilitation was made at the emergency department, by registered nurses with geriatric expertise. The assessment results were communicated to the hospital ward and to a municipal case manager (registered nurse). The case manager co-ordinated the planning for discharge together with a municipal interprofessional project team (social worker, physiotherapist and/or occupational therapist), hospital professionals and the older person. If approved by the older person, the case manager contacted the relatives, to inform/involve them in the care planning and to offer support and advice. Together with the municipal interprofessional project team, the case manager held a care-planning meeting in the older person’s own home, a few days after discharge. The meeting took place regardless of whether or not the older person needed home-care services.

Parallel with the new model, usual care was provided. It did not include any of the components inherent in the model. The care-planning meeting/discharge planning took place at hospital if new home-care services were needed, which was the usual routine. These meetings were run by the regular municipal interprofessional team (social worker, municipal nurse, occupational therapist and/or physiotherapist) and hospital professionals.

### Sample and data collection

The empirical data in this paper consist of nineteen audio-recorded care-planning meetings. Ten of the meetings took place in older people’s homes (from the “Continuum of Care, From the Emergency Ward to Living at Home—Implementation and Evaluation of an Intervention for Frail Elderly People” intervention project) [44], while the other nine meetings were held on hospital wards prior to discharge (usual care). Data from the meetings were collected in 2009 and 2010. Older people aged ≥ 80 years or ≥ 65 years with a minimum of one chronic illness and a need for assistance in activities of daily living were consecutively included in this study. Older people with severe acute illness, dementia or severe cognitive impairment or people receiving palliative care were not included. The data were collected by the second author, who was present at the meetings but did not participate in the conversations. This research strategy enabled the direct study of the needs-assessment and decision-making practices and the way the influence of the older people was manifested during the care-planning meetings. Access to the care-planning meetings was organised by the respective team’s social workers. Oral and written informed consent was obtained from all older people participating in the study. The relatives and the professionals at the meetings were informed orally and asked to participate. The study was approved by the Regional Ethical Review Board in Gothenburg, Sweden, registration number 413-08.

### Data analysis

The audio taped care-planning meetings were transcribed verbatim in Swedish by a professional with extensive experience of research interview transcription. Transcription conventions in a broad transcription format capturing were used to characterise issues, features, structures and interactions of importance. The transcriptions were compared and validated against the tape-recordings independently by two of the authors (HB, AD) and transferred to NVivo 9 (QSR International, Doncaster, Australia), a qualitative research software programme designed to help users organise and analyse non-numerical data.

A qualitative content analysis was performed [46, 47], focusing on the issues that were initiated at the meetings and how these were managed. The analysis of the care-planning meetings deals with the way people talk in social practice, how they perform tasks and what ‘facts’ mean through speaking with each other [48].

Two analysis domains were recognised. The first domain was the management of care-planning meetings, which included the structure of and interaction in the meetings. The second domain was the meeting contents, including the initiatives (the issues initiated and by whom), discussions (following the initiated issues) and decisions (relating to the initiated issues). The analysis was performed in three steps. In the first step, the care-planning meetings in the homes and in hospital (usual care) were analysed separately. In this step data diversity was reduced to the relevant [47]. The analysis was initially performed using an inductive approach. The analysis process was driven by the specific research aims, where descriptive categories, close to the data, were gradually developed. Meaning units of the first domain were identified as being structure and interaction, condensed and merged into sub-categories and categories. In the analysis of the second domain, meaning units were first identified as being initiatives, discussions or decisions. The meaning units were condensed and labelled with codes, describing the contents. Similar codes were put together in sub-categories and categories. Table 1 shows an example of the analysis process.

In the second step, a comparison was made between findings from meetings at home and meetings in hospital, in order to identify similarities and dissimilarities between the two groups. In the third and final step, overlapping themes on a more abstract level, cutting across categories in both domains, were identified. The themes were discussed in relation to literature and prior knowledge [47]. Independent coding and comparisons were made by the first and second author and discussions took place in each step of the analysis. Regular discussions between all authors took place throughout the entire analysis process.

In addition, older people’s talking space at the meetings was measured, in order to obtain a rough measurement of their involvement in the discussions. The talking space was measured as a word count [28, 49]. To make data from the meetings comparable, talk by hospital professionals regarding information about the hospital stay, at the meetings in hospital, was not taken into account.

## Results

### Overall characteristics of the care-planning meetings

In Table 2, a description of the participants, duration of care-planning meetings and older people’s talking space is presented. Older people’s median talking space was 40 per cent at the meetings at home and 20 per cent at the meetings in hospital.

An overview of domains, categories and sub-categories is presented in Table 3.

The categories were similar in meetings at home and in hospital. In some of the sub-categories there were dissimilarities, which are described under each heading. Overlapping themes, common to meetings at home and in hospital, are described at the end of the result section.

### Management of care-planning meetings

The management of the meetings constitutes the ‘framework’ for the older people’s opportunities for involvement in and influence over their future care and services.

#### Agenda for the meeting

The *overall meeting structures* varied between meetings at home and in hospital. At the home meetings there was no appointed chairperson, but most often the social worker and case manager alternated in leading the conversation. The issues at the meetings appeared to emerge spontaneously and there was no fixed order for speaking. At the meetings in hospital, the ward nurse was initially the chairperson, informing the municipal staff about the hospital stay, often in a dialogue with the older person. The social worker then took over and led the rest. Both at home and in hospital, the case manager or the social worker formulated a conclusion at the meeting’s conclusion.

The participating professionals initiated predefined *profession-specific topics.* The social worker asked about the need for help with household chores or personal care, the nurse asked about the health situation and medication, the physiotherapist asked about moving and the occupational therapist asked about activities of daily life. In the excerpt below, the case manager (a registered nurse) assessed a man’s foot pain problems.

Man, aged 74: “Well, it started because my shoes were too tight, so the skin cracked and it went on from there”.

Case manager: “Have you experienced any loss of feeling in it?”.

Man: “Right now, I have, but it sometimes comes back to life… and then it hurts. So it isn’t just a loss of feeling, there’s something wrong with the circulation. There’s no avoiding that”.

Case manager: “Does it hurt when you’re lying down?”.

Man: “Yes, sometimes”.

(Care-planning meeting at home, case 9)

#### Communication style

One important aspect of the meetings, which could have an impact on the older people’s influence, was the *fragmentary talk flow* at the meetings with a large number of interruptions. This often occurred when a professional suddenly started to ask the older person something before the previous topic had been completed, or when a person entered the room. These uncompleted discussions often resulted in repetitions of topics that had been initiated earlier. In this meeting at hospital, the social worker was interrupted by one of the nurses.

Social worker: “To enable everyone to do what they are expected to do, rehab here would like you to go home on Wednesday the 16th. Do you think that will be OK?”.

Woman, aged 83: “Yes, that’s fine for me—to my home?”.

Social worker: “Yes, you’ll go home on the 16th at about what time—two or one o’clock or when?”.

Ward nurse: “Do you have pharmacy-packaged medication?”.

Municipal nurse: “No, she doesn’t. I think she can manage that herself—with her medicines”.

(Care-planning meeting in hospital, case 4)

Most of the conversations at the meetings were directed at the older person. In spite of this, we identified internal talk between the professionals, where the older person was not involved. Furthermore, parallel talking was a phenomenon identified at the hospital meetings but rarely at home. This occurred when a specific professional from the municipality talked to a professional colleague at the hospital, simultaneously with something else being discussed at the meeting. Parallel talking at hospital meetings contributed to a fragmentary talk flow.

The meetings were characterised by a *friendly atmosphere*. On many occasions, the professionals used inviting questions, like asking the older people if there was something else they wanted to discuss. The *degree of everyday language* was fairly high at the meetings, while the professionals generally used a common informal language. In spite of this, the professionals occasionally used technical or medical terms. This occurred especially in the meetings at hospital.

#### Communication strategy

Different strategies used by the professionals in order to achieve their goals emerged at the meetings. The professionals frequently used a *negotiating* strategy, especially when they wanted the older person to accept a specific kind of help. In many cases the professionals cooperated to find a solution that they thought would be appropriate for the older person. The physiotherapist suggested that a woman should go to a day rehabilitation centre, but she was hesitant. The social worker supported the physiotherapist’s suggestion and together they negotiated with the woman.

Physiotherapist, municipality: “Would you like to think about it or shall I tell them to contact you?”.

Woman, aged 75: “No, I’d like to think about it”.

Physiotherapist, municipality: “OK”.

Woman: “I suppose the waiting times are not that long?”.

Physiotherapist, municipality: “Well, they can actually vary”.

Woman: “I see”.

Social worker: “So you can still refuse, even if you have said that you are interested”.

Physiotherapist, municipality: “Definitely, or you can go there and see what it’s like”.

(Care-planning meeting at home, case 5)

The professionals and/or the relatives also used a *persuasive* strategy, particularly when they wanted the older person to receive help. Both the relatives and the professionals used a *supportive* strategy. The relatives frequently emphasised the older people’s wishes or talked on their behalf.

### Content of care-planning meetings

The content of the care-planning meetings relates to issues initiated and discussed at the meetings and the corresponding decisions, taken by the older people, the relatives and the professionals. It reflects the area of possible issues for the involvement and influence of the older people.

#### Life situation and needs of older people

Most topics relating to the older people’s life situation and needs were introduced by the professionals, in accordance with their respective area of expertise, and formed part of their needs-assessment process. Issues relating to the older people’s *social situations,* including living circumstances, family situation and everyday life, were often initiated by the social worker but sometimes by other professionals, the older people or relatives. Concerns about *activities,* such as questions about how the older people managed to shower, dress, move and go out, were mostly initiated by the occupational therapist or the physiotherapist. In many cases, issues related to *physical health* and *mental health* were initiated by the older people or their relatives. The nurses and the case manager particularly initiated questions about physical and mental health, such as when they asked questions about pain and sleeping.

*Existential issues,* such as death, meaningfulness and loneliness, were introduced by the older people. One man hesitated about a possible move to a nursing home.

Man, aged 93: “It’s very difficult for me. If I leave my apartment, I’ll soon die. Because I won’t have anything left to like”.

Social worker: “Well, we’ll have to make sure that things are OK at home. We have to do that”.

Man: “That’s why it’s so incredibly important that you enjoy your life, isn’t it?”.

(Care-planning meeting in hospital, case 6)

The older people initiated conversations about their *life stories*, which could involve their earlier occupations and hobbies. Sometimes the professionals and the social workers in particular also asked them about these things.

#### Care and service

The initiatives and discussions about the older people’s life situation and needs resulted in questions about the care and services they needed. The professionals presented the municipality’s available range of care and services to the older people. *Home care* included home-help services, home nursing care, home rehabilitation and technical aids, while *service* included a cleaning service, laundry service and economic support. A large amount of time was spent on the professionals’ investigation of the current home care and service, the number of visits they had every day from the home-care team, what they actually did and how it worked. The professionals also initiated discussions about the need for future home care and service*.* All the professionals, the older people and the relatives were involved in these discussions. At the meetings at hospital, hospital staff also had opinions about future home care and service. The older people could choose from the available home care and services and say how much and how often they wanted to have this help.

The case manager or the municipal nurse initiated discussions about *medical treatment and medication,* including doctor’s visits (at home or at the clinic), and the dispensing and effects of drugs. The case manager in particular also gave nursing advice on how to treat different symptoms, such as breathing problems or swollen legs. The older persons and their relatives often had questions about medical treatment and medication, such as the routines for drug prescription and dispensing and how to obtain medical aids like compression stockings.

In the meetings in hospital, the older people’s *hospital stay and discharge* was discussed in the introductory phase, which included the transfer of information from hospital staff to municipal staff. The information concerned the reason for the hospital stay, care and rehabilitation during the stay and planning for discharge.

The older people’s* degree of satisfaction* emerged at the meetings. Satisfaction was frequently expressed in terms of gratitude for the opportunity to receive home care and the fact that the staff were kind. If the professionals asked the older person what he or she thought about the home care or service, he/she generally gave a fairly neutral answer. Others expressed dissatisfaction, such as a woman who complained about the many different staff members who helped her.

Woman, aged 77: “...lots of people came and said ‘My name is this or that and I come from here or there’. Right”.

Social worker: “The important thing is that you feel secure at home, that you can manage your daily life, that’s fine. You’re the one who says what you want and...”.

Woman: “But I’m not always sure that this is what happens”.

Social worker: “Really? Well you can in this case”.

(Care-planning meeting at home, case 10)

During the discussions about home care and services, the professionals explained the organisational structures, routines and rules, constituting *limits* reducing access to care or service for the older people. This man wanted to have the cleaning service temporarily for one month, until he finished his rehabilitation.

Social worker: “It won’t be the same people who come and help you with your compression stocking and training and who come and clean and wash. If you want that, you have to choose a company. We have private providers who do that work”.

Man, aged 80: “Hmm”.

Social worker: “It takes a week or two for things to start working. You have to start by choosing a company”.

Man: “There’s that...”.

Social worker: “And then they have to come here and meet you and agree on times and then it will take several weeks”.

Man: “I don’t want to be difficult, but wouldn’t it be easier to find someone on the web or in a newspaper who would come here and clean—if I’m honest”.

(Care-planning meeting at home, case 8)

#### Decisions

It often happened that the older people decided to *accept or reject* an offer of a specific kind of help. In other cases, the professionals or the older people and their relatives suggested that they should *wait and see,* because they thought it was too early to make decisions. However, most discussions ended in decisions by the professionals about the *same care and service* as before or *new care and service.* In the meetings at home, some decisions included immediate *action,* such as when the occupational therapist investigated the electrical backboard on a person’s bed, during a meeting. The professionals also promised to check, follow up or arrange something later. Other future action included forwarding information or offering help, if needed later.

Formulations in the decisions could be vague, resulting in *unclear* meanings of *decisions* or unclear responsibility*.* This occurred especially when there was more than one option in the decision. *Non-decisions* occurred when initiatives and discussions did not end in any decision at all. The topics that resulted in non-decisions were mostly initiated by the older people and related to health, existential issues or issues associated with organisational routines/limits. The non-decisions occurred in different forms: needs assessment not fulfilled, no solution found or no response. This man complained about the variation in the time of visits from the home-help service, but the professionals did not fulfill the assessment of his real concerns.

Man, aged 84: “I usually get up at 6 am, to be on the safe side. I get dressed, but they perhaps don’t come until 9.30 am”.

Social worker: “But do you want us to continue with the visits you currently have? That they come in the morning, afternoon and evening and…?”.

Man: “Yes, that’s…”.

Social worker: “You think that’s OK…”.

All: “Mmm, aaa, mmm…”.

Social worker: “And has the residential service with cleaning and so on started and does it work?”.

(Care-planning meeting at home, case 1)

### Themes identified at the care-planning meetings

At an overall level, we identified two themes that were common to all the care-planning meetings: balancing the focus of the care-planning meeting and enabling and obstructive conditions.

#### Balancing the focus of the care-planning meeting

The professionals steered the meetings towards predefined issues. When initiatives on the part of the older people were outside the professionals’ predefined agenda or did not match available help, they were not taken into consideration or the discussions were not completed. This frequently resulted in non-decisions. As the professionals’ task is to guarantee that the older people receive help, they need to focus on ‘possible’ issues. The negotiations used by the professionals often appeared when they tried to steer focus to the kind of help that was available. However, sometimes the change of focus was more unconscious as each professional was eager to talk about his/her ‘own’ issues and they interrupted a discussion that had not yet been completed. This led to a fragmentary talk flow, which may have caused the ‘loss of the thread’ for the older people and the other parties involved. In turn, it may have obstructed a comprehensive understanding of the older people’s needs, as well as their opportunities for active involvement and influence.

#### Enabling and obstructive conditions

The older people’s influence at the care-planning meetings was affected by the conditions surrounding these meetings. Enabling conditions were the availability of extensive and frequent help corresponding to the basic needs of the older people. The older people could choose in a fairly unrestricted way both the care and service they wanted and the frequency of this help within the help available in the municipality. For some services, they could also choose the provider of the specific services. Planning care at home appeared to be an enabling condition for older people’s involvement in the discussions, as their median talking space was 40 per cent compared with 20 per cent at meetings in hospital and there was hardly any parallel talking in meetings at home. Other conditions obstructed the older people’s influence, regardless of whether the meeting was held at home or in hospital. Organisational guidelines restricted the professionals’ ability to modify the help according to individual wishes. In these cases non-decisions appeared or a negotiation strategy was used by the professionals. The older people were unable to influence how care and services would be performed or organised, not the time of a visit nor the number of home care staff providing the help.

## Discussion

It was noted that older people’s median talking space was 40 per cent at the home meetings and 20 per cent at the hospital meetings. This may indicate a more active involvement on the part of the older people in the home meetings. Fewer people participated in the home meetings and there was much less parallel talking, which probably made it easier for the older people to follow and get involved in the discussions. On the other hand this study shows that involvement in the discussions does not guarantee older people’s influence. The older people had some influence over concerns relating to the amount of care and service and the choice of provider. The professionals at the meetings were, however, not able to make decisions about how the help would be provided or organised and they appeared to be bound by the rules of the organisation. Within negotiation processes, solutions were developed in many cases which could be acceptable to both older people and professionals.

The professionals’ communication domination found in our study confirms findings from earlier research [27, 28]. The professionals attempted to steer the focus of the meetings to their own agenda and showed a lack of preparedness for dealing with ‘unexpected questions’ initiated by the older people, such as issues about the way delivery of care was organised or issues of an existential nature. The older people were unable to influence the “hidden” agenda and were probably unaware of the topics they were expected to talk about. The professionals initiated most of the conversation, but they also invited the older people to bring up their concerns. Despite this, the initiatives that fell outside the professionals’ predefined agenda were not taken into consideration or the discussions were not completed. As a result, the professionals, as institutional representatives, controlled the discussions and proposed solutions [14, 50, 51].

Our results confirm that older people’s needs, as well as corresponding care and services, are classified for management in predefined categories, typical of processing procedures in human service organisations [37, 52]. This study reveals that organisational rules inhibit the older people’s influence over needs assessment and decisions relating to care and service, which has also been found in other studies in this field [10, 14, 18, 19]. Sullivan [14] showed that professionals attempted to direct needs assessment to secure an outcome that was right for the community care system. Furthermore behaviour rules [41] and the professionals’ social frames in the meetings “defined” the older person and determined the outcome [14]. Conversely, the professionals used a negotiation strategy to find practicable solutions. In earlier studies it was concluded that professionals used negotiation with the intention of finding solutions allowing the professionals to make sure that the basic needs of the older people were met and that they received the necessary help [18, 40]. We also observed that the professionals in many cases cooperated with each other when they negotiated with the older person, which increased the professionals’ power. This may be seen as forming alliances to convince the older person to accept the professionals’ definition of the situation [35, 36].

The professionals have limited power to make individual solutions, as their power is mainly rooted in the organisation [53, 54]. This results in difficulties for the professionals to empower the older people to influence the needs assessment and decision-making [13]. The organisational obstacles appear to be inherent in the care-planning system and the location of care-planning meetings had no major impact on the older people’s influence. During the meetings, some of the older people mentioned experiences of expressing dissatisfaction with services. They questioned the professionals’ statements giving them the right to do so, implying that their prospects of real influence were restricted. Instead, in many cases, the older people accepted the help they could get. The older people’s decisions to accept or comply with the help they could get may reflect the power of the professionals and the organisation over the older people and older people’s conditions of dependence [38, 39]. In other cases, if the older people had opportunities to manage without this help, they abstained from help they did not regard as meeting their needs, which is also described by Hardy et al*.* [9] and Starkey [43].

As concluded in other studies [9, 40], our results indicate that older people’s influence is mostly limited to the negative choice of refusing care and services. In the care-planning meetings, the professionals were unable to meet special requirements or wishes beyond the existing range of care and services [9, 10, 12]. This is also confirmed by another study, showing the standardised nature of available help and the fact that older people perceived no actual influence over the decisions about their home help [30].

### Methodological considerations

It might be a disadvantage that the original spoken language (Swedish) was translated into English. However, our analyses were carried out on the Swedish versions. Here, we present data in approximate English translations, and our discussion refers to points in the translated data.

Authentic audio recordings have both strengths and weaknesses. They allowed us to study care-planning meetings as they occur in their natural settings. Care-planning meetings represent a common case of the individual organisational interface and provide an excellent opportunity for direct study of these practices. On the other hand, they inhibited our opportunity to receive knowledge about the way the older people perceived their influence. This would have required interviews, which was beyond the scope of this study. The presence of a researcher at the meetings may have impacted interactions that took place. In all probability, however, this did not have any negative effects for the older people, as it is reasonable to believe that the participating professionals did their utmost to act as correctly as possible and to make a good impression [55].

Moreover, some of the older people in the home meetings appeared to be less frail than those at the hospital meetings. This was a result of the design of the project, as care-planning meetings at home were held for all participants, while care-planning meetings at hospital were only held if new home help was needed. This probably had no major impact on our results, as all the older people belonged to the study’s target group.

## Conclusion

The results of this study call for further attention to older people’s influence and professionals’ opportunities to empower the older people within the context of the care-planning system in health and social care organisations. Our findings indicate a need for continuing discussions about the professionals’ opportunities to make decisions according to their professional assessments that go beyond the standard range of care services and tailor individual care packages. The current policy orientation, emphasising a consumerist approach to empowerment and the users’ choice between different providers of care, risks disregarding important aspects of older people’s influence. The decisions made at care-planning meetings have a great impact on older peoples’ everyday lives: there is a need to consider how older people’s opportunities to influence the day-to-day provision of care and service can be improved. The way in which care planning is organised appears to shape the conditions for the involvement of older people. Planning care at home was shown to have positive tendencies regarding older people’s involvement in the discussions at the meetings, but it did not lead to any real influence. Additional efforts at both professional and structural level may be required in order to obtain any real influence for older people. Therefore we suggest focusing on organizational strategies, professional discretion and joint care planning to further improve integrated care for older people.

## Reviewers

**Elisabet Cedersund,** Professor in Ageing and Later Life, Department of Social and Welfare Studies Linköping university, SE-60174 Norrköping, Sweden

**Mary Pat Sullivan**, PhD, MSW, BSW, Senior Lecturer, School of Health Sciences and Social Care; Programme Director, Gerontological Social Work Research, Brunel Institute for Ageing Studies, Mary Seacole Building, Brunel University

One anonymous reviewer

## Figures and Tables

**Table 1.**
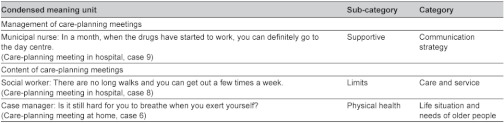
Description of condensed meaning units, sub-categories and categories in the analysis process of the two domains

**Table 2.**
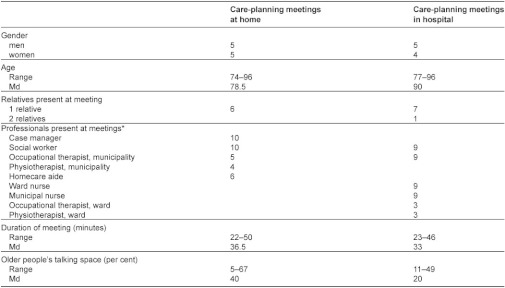
Participants, duration of care-planning meetings and older people’s talking space

Values are numbers unless stated otherwise. *Professionals in total per meeting: 2–4 at home and 4–6 in hospital.

**Table 3.**
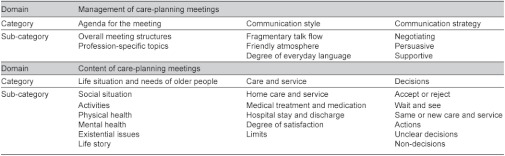
Overview of domains, categories and sub-categories
